# Development and validation of the Chinese university students’ social mentality questionnaire: a four-component model study

**DOI:** 10.3389/fpsyg.2026.1804407

**Published:** 2026-06-24

**Authors:** Hui Zhang, Rui Guo, Xiao Yang, Kaida Wang

**Affiliations:** 1 School of Marxism, Zhejiang University, Hangzhou, China; 2 Mental Health Education and Counseling Center, Zhejiang University, Hangzhou, China; 3 School of Marxism, Hangzhou Normal University, Hangzhou, China; 4 School of Economics, Hangzhou Normal University, Hangzhou, China

**Keywords:** Chinese university students, four-component model, psychometric properties, scale development, social mentality

## Abstract

**Background:**

Social mentality serves as a crucial psychological barometer of social changes. However, there is a lack of systematic, multidimensional measurement tools specifically tailored for Chinese university students, a group critical to the nation’s future. Based on the theoretical framework of the Cognition-Emotion-Values-Behavior four-component model, this study aimed to develop and validate the Chinese University Students’ Social Mentality Questionnaire (CUSSMQ).

**Methods:**

A total of 1,315 university students from 10 universities across China were recruited. The sample was randomly split into two independent subsamples for Exploratory Factor Analysis (EFA, *N* = 658) and Confirmatory Factor Analysis (CFA, *N* = 657). Item analysis, internal consistency reliability testing, and criterion-related validity testing were conducted to evaluate the psychometric properties of the scale.

**Results:**

EFA results identified a stable factor structure across four subscales: (1) Social Cognition (6 factors, including distinct Family Support and Social Support); (2) Social Emotion (3 factors, identifying Positive, Oppositional, and Vulnerable emotions); (3) Social Values (initially 4 factors in EFA; Materialism was subsequently removed at the CFA stage due to a non-significant higher-order loading, yielding 3 factors: National Identity, Pragmatism, and Social Responsibility); and (4) Social Behavioral Tendencies (3 factors). CFA indicated excellent model fit for all subscales (CFI ≥ 0.929, TLI ≥ 0.923, RMSEA ≤ 0.068, SRMR ≤ 0.067). The scale demonstrated high internal consistency, with Cronbach’s *α* coefficients ranging from 0.72 to 0.95 across dimensions. Criterion-related validity was supported by significant correlations between the CUSSMQ dimensions and prosocial tendencies.

**Conclusion:**

The CUSSMQ proves to be a reliable and valid instrument with sound psychometric properties. Its refined dimensional structure—particularly the differentiation in support sources and emotional types—provides a solid empirical foundation for educators and policymakers to monitor and guide the social mentality of Chinese youth in a targeted manner.

## 1 Introduction

Social mentality serves as a comprehensive psychological projection of social structural transformation and civilization development. It represents the psychological characteristics and mental states shared by the majority of society members regarding the macro-social environment, social relationships, and their own situations (Wang, 2023; Yang, 2006). Distinct from the biopsychosocial evolutionary perspective which views social mentalities as interpersonal motivational systems (Gilbert, 1989, 2005; Brasini et al., 2020), the current study adopts a macro-sociological framework specific to the Chinese context. Scientific monitoring and positive guidance of social mentality are not only integral to the modernization of social governance (Ding, 1996) but also crucial for identifying social risks and forging social consensus. Critically, although social mentality is a group-level concept, it is constituted by the psychological characteristics of individual members. Following the individual-referent composition model (Chan, 1998; Klein and Kozlowski, 2000), when individual-level cognitions, emotions, values, and behavioral tendencies exhibit sufficient convergence across a population, they constitute social mentality at the collective level. This compositional perspective provides the methodological foundation for the present study: we measure individual-level components of social mentality using established psychological constructs, while the distributional patterns of these responses across the sample reflect the macro-level social mentality of the group.

As a group shouldering the responsibility for the nation’s future, university students’ social mentality directly reflects the interaction between the younger generation and social reality. Their psychological state has profound implications for both individual healthy development and long-term social stability (Yu and Liu, 2014). However, contemporary Chinese society is undergoing unprecedented rapid transformation. As noted by Cai et al. (2020) and Xu and Hamamura (2014), widely-held values are shifting toward a complex coexistence of rising individualism and persisting traditional collectivism, accompanied by a general decline in social trust.

Specifically, today’s university students are situated in a unique dual context of being “Digital Natives” and living in the “Post-pandemic Era.” Longitudinal studies indicate that major public health crises (e.g., COVID-19) have caused significant fluctuations in students’ social mentality (Zhao et al., 2022). Furthermore, facing intense academic and employment competition (often termed “Involution”), some youths have adopted passive coping strategies such as “Lying flat” (He et al., 2025). These phenomena underscore the emergence of distinct social cognitive and emotional patterns that traditional measurement tools—often designed for the general public—fail to capture accurately.

Consequently, there is an urgent need for a specialized instrument tailored to this demographic. Drawing on the authoritative “Cognition-Emotion-Values-Behavior” four-component model (Ma, 2008), this study aims to develop and validate the Chinese University Students’ Social Mentality Questionnaire (CUSSMQ). By integrating theoretical rigor with contemporary relevance, this study seeks to provide a psychometrically sound tool for educators and policymakers to deeply understand, scientifically assess, and effectively nurture a positive social mentality among the new generation of university students.

### 1.1 The need for a tailored instrument

A review of existing measurement efforts further highlights the gap that the present study aims to fill. Within the Chinese sociological tradition, Yang (2006) provided an influential theoretical definition of social mentality but did not develop a corresponding measurement instrument. Wang and Pan (2013) constructed a social mentality scale; however, it was designed for the general public, and its dimensional structure does not encompass behavioral tendencies—a component considered essential under the four-component framework. Lv et al. (2019) developed a social mentality scale specific to the doctor-patient relationship context, which lacks generalizability to broader populations. In the Western literature, Brasini et al. (2020) published the Social Mentalities Scale based on Gilbert’s (1989, 2005) biopsychosocial evolutionary perspective, which conceptualizes social mentalities as interpersonal motivational systems—a theoretical orientation fundamentally different from the macro-sociological framework adopted in the present study. Notably, none of these instruments were specifically developed for university students, a population whose unique developmental stage, digital immersion, and exposure to intense social competition demand tailored measurement. This absence of a dedicated, theoretically grounded, and psychometrically validated instrument for Chinese university students’ social mentality constitutes the primary research gap that the present study seeks to address.

### 1.2 The four-component theoretical framework

To systematically capture the “micro-ecology” of Chinese university students, this study adopts the four-component model of social mentality (Ma, 2008; Wang, 2013). While previous models and scales provided a foundation (e.g., Wang and Pan, 2013; Yang, 2006), the four-component framework offers a superior structural advantage by integrating Social Cognition, Social Emotion, Social Values, and Social Behavioral Tendencies into a coherent “Input-Process-Output” feedback loop. This model has been widely validated in the Chinese context (e.g., Ma, 2011; Lv et al., 2019) and effectively bridges the gap between psychological mapping and behavioral willingness.

### 1.3 Construct operationalization

Based on this framework, and consistent with the individual-referent composition approach outlined above, we constructed the indicator system by operationalizing each component at the individual level, synthesizing classical psychological theories with the specific cultural context of contemporary China.

#### 1.3.1 Social cognition

Social cognition refers to individual-level processing of information regarding the self and social reality. Drawing on Social Cognitive Theory (Bandura, 1986, 2001) and Social Capital Theory (Putnam, 2000), we operationalized this component into three dimensions: Subjectivity: Based on Bandura’s (2001) agentic perspective, this dimension reflects students’ self-efficacy and belief in their ability to influence social processes, serving as a buffer against “learned helplessness.” Social Connectivity: This reflects the depth of social embedding. Incorporating Putnam’s (2000) concept of reciprocity and Cohen and Wills’ (1985) buffering hypothesis, we measure Social Trust and Social Support (Yang and Han, 2021). Crucially, given the “family-oriented” nature of Chinese culture (Xin and Su, 2020), we explicitly separated Family Support from general interpersonal support to highlight its unique protective role. Social Output Evaluation: Grounded in Diener’s (1984) subjective well-being model, Social Satisfaction was operationalized by adapting the five items from the Satisfaction with Life Scale (SWLS; Diener et al., 1985). Although the SWLS was originally designed to measure global life satisfaction, its items capture individuals’ cognitive appraisals of whether their life conditions meet their expectations—a judgment process inherently shaped by the social environment. In the context of the present study, university students’ satisfaction with life is substantially influenced by social factors such as perceived fairness, opportunity structures, and social support systems. We therefore adopted the SWLS items as indicators of Social Satisfaction, reflecting the degree to which the social environment meets students’ fundamental expectations and needs.

#### 1.3.2 Social emotion

Social emotion represents the “affective tone” of social mentality. Guided by the Circumplex Model of Affect (Russell, 1980) and the Positive–Negative Affect model (Watson et al., 1988; Qiu et al., 2008), we parsed social emotions based on their adaptive functions: Constructive Positive Emotion: Drawing on Fredrickson’s (2001) Broaden-and-Build theory, this reflects the enthusiasm and vitality necessary for social exploration. Defensive Negative Emotion: Unlike generic negativity, we differentiated this into inward-directed Vulnerable Emotion (e.g., anxiety, referencing digital “FoMO”; Wang and Zhang, 2024) and outward-directed Oppositional Emotion (e.g., hostility). This distinction captures the diverse emotional responses—ranging from “lying flat” to social conflict—triggered by stress.

#### 1.3.3 Social values

Social values serve as the core motivational drivers. Based on Rokeach’s (1973) value theory and Hofstede’s (1980) cultural dimensions, we examined the tension between collective and individual orientations: Collective Orientation: Rooted in Social Identity Theory (Tajfel and Turner, 1979) and civic virtue traditions (Berman, 1997), this includes National Identity and Social Responsibility, reflecting the students’ commitment to the broader community. Individual Orientation: This captures survival strategies in a competitive society. We distinguished between Pragmatism (prioritizing utility, echoing Dewey, 1920) and Materialism. Following Richins and Dawson (1992), Materialism is defined here as viewing possessions as signals of social status, a potentially maladaptive response to status anxiety.

#### 1.3.4 Social behavioral tendencies

Behavioral tendency is the proximal predictor of action (Ajzen, 1991). We focused on three prosocial modalities: Altruistic Behavior: Based on the Empathy-Altruism hypothesis (Batson, 1987), focusing on voluntary help at the micro-interpersonal level. Public Participation: Drawing on the Civic Voluntarism Model (Verba et al., 1995), assessing engagement in macro-public affairs. Conflict Resolution: Based on Rahim’s (1983) conflict handling styles, focusing on rational and integrative approaches to social friction, which indicates the maturity of social mentality.

## 2 Methods

### 2.1 Participants

A total of 1,413 university students were initially recruited from 10 universities across China using a convenience sampling method. The 10 universities were purposefully selected to ensure diversity in both geographic distribution (covering eastern, central, and western regions of China) and institutional type (including 7 Double First-Class universities, 2 regular undergraduate universities, and 1 vocational colleges). Data collection was conducted from May 7 to June 30, 2025, via Wenjuanxing, a widely used online survey platform in China. After excluding invalid responses based on predefined quality control criteria (see Section 2.3), a total of 1,315 valid questionnaires were retained, yielding an effective response rate of 93.13%. The final sample consisted of 546 males (41.5%) and 769 females (58.5%). Regarding grade level, the sample included 363 freshmen (27.6%), 234 sophomores (17.8%), 231 juniors (17.6%), 209 seniors (15.9%), and 278 postgraduate students (21.1%). Participants were predominantly majoring in humanities and social sciences (46.8%), followed by science and engineering (46.2%) and other fields (7.0%).

To establish and validate the factor structure of the CUSSMQ, the total sample (*N* = 1,315) was randomly split into two independent subsamples using a computer-generated randomization sequence. Sample 1 (*n* = 658) was used for Exploratory Factor Analysis (EFA) to identify the initial factor structure. Sample 2 (*n* = 657) was used for Confirmatory Factor Analysis (CFA) to cross-validate the model fit. Chi-square tests and *t*-tests confirmed that there were no significant differences in demographic characteristics (gender and grade) between the two subsamples (*p* > 0.05), ensuring that the split was unbiased (see Table 1).

**Table 1:** Demographic characteristics of the total sample and subsamples.

Characteristic		Sample 1		Sample 2	
		*N*	%	*N*	%
Total		658	100%	657	100%
Gender	Male	272	41.3%	274	41.7%
	Female	386	58.7%	383	58.3%
Grade	First-year undergraduate	179	27.2%	184	28.0%
	Second-year undergraduate	118	17.9%	116	17.7%
	Third-year undergraduate	109	16.6%	122	18.6%
	Fourth-year undergraduate	108	16.4%	101	15.4%
	Postgraduate	144	21.9%	134	20.4%

### 2.2 Instrument development

#### 2.2.1 Item generation

The initial item pool was generated using a deductive approach. Grounded in the “Cognition-Emotion-Values-Behavior” four-component model proposed by Ma (2008), we conducted an extensive literature review to operationalize each dimension based on established theoretical frameworks and existing scales.

It is important to clarify why new items were developed rather than directly adopting existing validated instruments. Our rationale rests on three considerations. First, from the perspective of systematic integration, existing instruments for constructs such as social trust (e.g., Putnam, 2000), affect (e.g., Watson et al., 1988), and values (e.g., Schwartz, 1992) were each developed independently for their respective single constructs. These instruments differ in their theoretical frameworks, scoring systems, and scale anchors, making direct combination impractical. The core value of the present study lies in integrating all four components within a unified “social mentality” theoretical framework, ensuring that all dimensions operate under a coherent measurement logic to effectively assess this umbrella construct. Second, from the perspective of cultural contextualization, most established instruments were developed and validated with Western samples, and direct transplantation poses cultural adaptation risks. The present study adapted and generated items specifically for Chinese university students, and the EFA results empirically validated this approach—for example, Family Support emerged as a factor distinct from general Social Support, reflecting the unique protective role of familial bonds in Chinese culture (Xin and Su, 2020), and the differentiation between Materialism and Pragmatism captured distinct value orientations among contemporary Chinese youth under competitive pressure. Third, from the perspective of psychometric screening, although items were informed by established instruments, not all items from those instruments are suitable for measuring the specific construct of “social mentality.” Through rigorous item analysis, EFA, and CFA procedures, the present study screened, refined, and reorganized an initial pool of 117 items into a set of 69 items, empirically validating their effectiveness within the hierarchical structure of the CUSSMQ (1 higher-order factor → 4 s-order factors → 15 first-order factors).

*For Social Cognition*, items measuring social efficacy were developed based on Bandura’s (2001) Social Cognitive Theory. Items assessing social trust and social support were derived from Putnam’s (2000) Social Capital Theory. Additionally, items related to social satisfaction were adapted from the Satisfaction with Life Scale (SWLS; Diener et al., 1985), and items on social safety were developed based on Lind and Tyler’s (1988) procedural justice perspective.

*For Social Emotion*, drawing on the Circumplex Model of Affect (Russell, 1980) and the Positive and Negative Affect Schedule (PANAS; Watson et al., 1988), we generated items to capture both constructive positive emotions (e.g., joy, pride) and defensive negative emotions (e.g., anxiety, hostility).

*For Social Values*, reflecting the tension between collective and individual orientations in transitional China, items were constructed based on Rokeach’s (1973) value theory and Hofstede’s (1980) cultural dimensions. Specifically, materialism items were adapted from Richins and Dawson (1992), while pragmatism items were developed referencing Dewey’s (1920) instrumentalism.

*For Social Behavioral Tendencies*, guided by the Theory of Planned Behavior (Ajzen, 1991), items measuring altruistic behavior, public participation, and conflict resolution were adapted from the Prosocial Tendencies Measure (Carlo and Randall, 2002) and the Civic Voluntarism Model (Verba et al., 1995).

All generated items were reviewed by the research team to ensure linguistic clarity and cultural appropriateness for Chinese university students. This process resulted in an initial pool of 117 items, rated on a 5-point Likert scale (1 = strongly disagree/never to 5 = strongly agree/always).

#### 2.2.2 Measures

The Chinese University Students’ Social Mentality Questionnaire (CUSSMQ) The final version of the CUSSMQ, developed in this study, consists of 69 items across four subscales: Social Cognition, Social Emotion, Social Values, and Social Behavioral Tendencies. Participants rated items on a 5-point Likert scale. The anchor points varied slightly by dimension to ensure semantic appropriateness: for Social Cognition and Social Values, the scale ranged from 1 (strongly disagree/very distrustful) to 5 (strongly agree/very trustful); for Social Emotion and Behavioral Tendencies, it ranged from 1 (never/very unwilling) to 5 (always/very willing). Negative items (e.g., those measuring Oppositional Emotion and Vulnerable Emotion) were reverse-coded so that higher scores generally reflect a more positive or adaptive social mentality, or higher levels of the specific construct being measured.

*Criterion Measure*: To assess criterion-related validity, the Prosocial Tendencies Measure (PTM), originally developed by Carlo and Randall (2002) and revised for the Chinese context by Kou et al. (2007), was employed. This scale consists of 26 items assessing six dimensions of prosocial behaviors: altruistic, compliant, emotional, public, anonymous, and emergency. Items are rated on a 5-point Likert scale ranging from 1 (does not describe me at all) to 5 (describes me greatly). The PTM is widely used in Chinese adolescent and young adult research. In the present study, the scale demonstrated good internal consistency, with Cronbach’s *α* coefficients for the six sub-dimensions ranging from 0.78 to 0.87.

### 2.3 Procedure

Ethical Approval and Informed Consent The study protocol was reviewed and approved by the Institutional Review Board (IRB) of the corresponding author’s affiliation (Approval No. 202503002). Prior to accessing the survey, all participants were presented with an electronic informed consent form outlining the study’s purpose, the confidentiality of the data, and their rights. Participants were explicitly informed that their participation was voluntary and anonymous, and that they could withdraw from the study at any time without consequence. Only those who clicked the “I agree to participate” button could proceed to the questionnaire.

Data Collection Data collection was conducted from May 7, 2025, to June 30, 2025, using Wenjuanxing, a secure online survey platform widely used in China. The survey link was converted into a QR code and distributed via major social media and communication platforms (e.g., WeChat and DingTalk) to university students across 10 institutions. Participants could complete the survey on mobile phones or computers at their convenience.

Survey Administration and Quality Control The questionnaire contained 117 items. To minimize potential order effects and response bias, the presentation order of the items was randomized for each participant. Rigorous quality control procedures were implemented to ensure data validity. Questionnaires were excluded if they met the following criteria: (1) insufficient completion time (responses with a duration of less than 300 s, considering the average completion time was approximately 10 min); or (2) regular response patterns (e.g., straight-lining or choosing the same option for all items). After data cleaning, a total of 1,315 valid questionnaires were retained, resulting in an effective response rate of 93.13%.

### 2.4 Data analysis

Data analysis was performed using SPSS 26.0. The analysis followed a five-step procedure. *Item Analysis*: The critical ratio method (*t*-test) and item-total correlations were used to screen the initial items. Items were removed if they failed to discriminate between high- and low-scoring groups or showed low correlations with their respective subscales. *Exploratory Factor Analysis (EFA)*: EFA was conducted on Sample 1 (*n* = 658) to determine the factorial structure. Principal Component Analysis (PCA) with Direct Oblimin rotation was employed. The retention of factors was guided by eigenvalues >1, the scree plot, and parallel analysis. Items were retained based on factor loadings (>0.50), absence of cross-loadings (>0.20 difference), and communalities (>0.30), item numbers less than 5 per factor. *Confirmatory Factor Analysis (CFA) and Convergent Validity*: CFA was conducted on Sample 2 (*n* = 657) using Maximum Likelihood estimation to test the model fit. Model fit was evaluated using $\chi^{2}$ /d*f* (<5), CFI (>0.90), TLI (>0.90), RMSEA (<0.08), and SRMR (<0.08) (Hu and Bentler, 1999). Furthermore, Convergent Validity was assessed by calculating the Average Variance Extracted (AVE) and Composite Reliability (CR) for each latent construct. *Discriminant Validity and Reliability*: Discriminant validity was examined using the Fornell-Larcker criterion, which requires the square root of the AVE for each construct to exceed its correlations with other constructs. Reliability was assessed using Cronbach’s *α* and split-half reliability coefficients. *Nomological Validity*: To assess the nomological validity of the instrument, we conducted a regularized partial correlation network analysis. The network was estimated using the Graphical Least Absolute Shrinkage and Selection Operator (gLASSO) with the Extended Bayesian Information Criterion (EBIC) as the model selection criterion. Node bridge strength was calculated to quantify how strongly each construct of the instrument connects differently with prosocial tendencies which is theoretically related with social mentality (Epskamp et al., 2018). *Common Method Bias Test*: Since data were collected via self-report measures, Harman’s single-factor test was conducted to check for common method bias. An unrotated factor analysis was performed on all items to ensure that no single factor accounted for the majority of the variance (threshold <40%).

## 3 Results

### 3.1 Common method bias and item analysis

*Common Method Bias Test*: Since all data were collected using self-report measures, Harman’s single-factor test was conducted to examine the potential influence of common method bias. An exploratory factor analysis (EFA) without rotation was performed on all 117 items. Results revealed 21 distinct factors with eigenvalues greater than 1. The first unrotated factor explained [22.85%] of the total variance, which was well below the critical threshold of 40% (Podsakoff et al., 2003). This indicates that common method bias is likely not a pervasive issue in the present study.

*Item Analysis*: Item analysis was conducted to evaluate the discriminatory power and homogeneity of the 117 initial items.

First, the critical ratio method was employed. The total scores of each subscale were ranked, and independent samples *t*-tests were conducted between the highest 27% and lowest 27% groups. Results showed that all items exhibited statistically significant differences between the two groups (*t*-values ranged from −24.50 to −2.76, *p* < 0.001), except for one item in the Social Behavioral Tendencies subscale (*t* = −0.05, *p* = 0.962), which failed to discriminate effectively.

Second, item-total correlations were examined. The correlation coefficients for the majority of items ranged from 0.31 to 0.77 (*p* < 0.001), indicating good internal consistency. However, the non-significant item identified in the *t*-test also showed a poor correlation (*r* = 0.02, *p* = 0.532). Additionally, three items in the Social Values subscale were removed due to low or negative correlations (*r* = 0.12, −0.30, −0.18), suggesting potential ambiguity or measurement issues. As a result of these analyses, 4 items were excluded. The remaining 113 items were retained for the subsequent Exploratory Factor Analysis.

### 3.2 Exploratory factor analysis (EFA results on sample 1)

Exploratory Factor Analysis (EFA) was conducted on Sample 1 (*n* = 658) to determine the underlying factor structure of the CUSSMQ subscales. The Kaiser-Meyer-Olkin (KMO) measures for the CUSSMQ was 0.93. Bartlett’s tests of sphericity were significant (*p* < 0.001), indicating excellent suitability for factor analysis. Following the approach of Li et al. (2024), we included all items in the exploratory factor analysis (EFA). The EFA resulted in 16 factors, explaining 71.82% of the total variance, with factor loadings presented in Table 2.

**Table 2:** Factor loadings and communalities of the CUSSMQ.

Items	Social support	Social trust	Social efficacy	Social safety	Social satisf.	Family support	Positive emotion	Oppositional emotion	Vulnerable emotion	National identity	Materialism	Pragmatism	Social responsibility	Altruistic behavior	Conflict resolution	Public participation	Communality
support6	**0.91**	0.09	−0.03	−0.07	−0.05	0.01	0.07	−0.03	0.03	−0.03	−0.01	0.01	−0.05	−0.04	0.05	−0.03	0.81
support7	**0.88**	0.02	−0.05	0.00	−0.01	0.02	0.07	0.00	0.02	0.01	0.01	0.01	0.02	0.01	0.00	−0.03	0.82
support4	**0.85**	0.03	0.05	0.04	−0.08	0.02	−0.06	0.10	−0.06	0.00	0.01	−0.04	−0.01	0.00	0.03	−0.01	0.73
support8	**0.79**	0.02	0.05	0.00	0.03	0.01	0.04	0.01	0.00	0.07	−0.01	0.00	0.01	−0.05	−0.04	0.02	0.72
support2	**0.73**	−0.02	0.10	0.04	0.16	0.01	−0.10	−0.06	0.04	−0.06	0.02	0.06	−0.06	0.02	−0.03	0.05	0.63
trust4	0.11	**0.88**	−0.08	−0.02	−0.04	−0.03	−0.05	−0.01	0.02	−0.04	−0.06	0.01	−0.02	0.05	0.05	−0.04	0.68
trust3	0.04	**0.85**	0.03	0.00	0.01	−0.02	0.01	0.02	0.01	−0.03	0.08	0.04	0.06	0.01	−0.01	−0.03	0.80
trust2	0.05	**0.81**	0.00	0.04	0.06	−0.02	0.01	0.03	0.00	0.04	0.01	−0.09	0.03	−0.02	0.00	0.03	0.79
trust5	−0.07	**0.79**	−0.03	0.03	0.08	−0.01	−0.04	−0.01	−0.01	−0.03	0.02	0.06	−0.04	−0.01	0.00	0.13	0.65
trust1	0.00	**0.71**	0.01	0.05	−0.04	0.03	0.00	0.03	0.00	0.17	0.04	−0.01	0.03	−0.01	−0.03	−0.01	0.69
efficacy3	0.02	−0.05	**0.94**	−0.02	0.00	−0.04	0.02	0.03	−0.01	−0.08	0.06	0.03	0.00	0.02	−0.02	0.01	0.80
efficacy2	0.03	0.00	**0.85**	0.01	−0.04	0.02	−0.05	−0.03	−0.04	−0.02	−0.05	−0.01	0.01	−0.04	0.05	0.05	0.79
efficacy1	−0.01	−0.04	**0.84**	0.01	0.08	−0.03	0.04	0.04	−0.03	0.05	−0.05	−0.09	−0.05	0.01	0.05	0.04	0.80
efficacy5	−0.01	−0.01	**0.83**	−0.04	0.06	−0.03	0.06	0.00	0.05	0.05	0.02	0.04	−0.01	−0.02	0.00	−0.02	0.71
efficacy7	0.08	0.04	**0.70**	0.04	−0.12	0.10	−0.12	−0.05	0.01	0.00	0.01	0.04	0.10	0.06	0.02	−0.08	0.60
safety7	−0.10	−0.04	−0.02	**0.85**	0.05	0.02	0.05	0.00	0.05	0.02	0.00	0.05	−0.02	0.01	0.04	−0.09	0.74
safety8	−0.08	0.04	−0.04	**0.83**	0.00	0.02	0.03	0.01	0.02	0.03	−0.01	0.05	−0.03	0.06	0.04	−0.08	0.74
safety1	0.03	0.00	0.06	**0.81**	−0.06	−0.07	−0.03	−0.04	−0.02	−0.07	−0.02	−0.09	0.03	−0.02	0.04	0.05	0.64
safety6	0.04	0.10	0.01	**0.76**	−0.03	0.07	−0.01	−0.02	−0.02	−0.05	0.01	0.00	0.09	−0.07	−0.04	0.05	0.70
safety3	0.13	0.00	−0.04	**0.75**	0.03	−0.07	0.05	0.08	−0.06	0.07	−0.02	−0.03	−0.07	0.01	−0.07	0.06	0.62
satisf.1	−0.02	−0.02	−0.04	−0.03	**0.92**	−0.01	−0.13	0.01	0.01	−0.07	−0.03	−0.03	−0.08	0.02	0.00	0.10	0.64
satisf.2	0.03	0.03	0.00	0.00	**0.90**	−0.06	0.00	0.03	0.05	−0.01	−0.01	0.02	−0.06	−0.01	−0.02	0.05	0.75
satisf.3	−0.03	0.08	0.01	0.03	**0.76**	0.06	0.00	−0.03	0.01	0.02	0.01	−0.04	0.05	−0.02	0.07	−0.09	0.75
satisf.4	0.02	0.00	0.03	0.01	**0.72**	0.04	0.10	−0.09	−0.02	0.04	−0.01	0.02	0.06	0.00	−0.02	−0.10	0.77
satisf.5	0.00	−0.01	0.07	0.02	**0.63**	0.07	0.12	−0.05	−0.01	0.05	0.00	0.03	0.10	0.04	−0.02	−0.14	0.73
family2	0.08	−0.06	−0.05	−0.04	−0.03	**0.93**	0.00	0.06	−0.01	0.05	0.02	0.03	0.02	−0.05	0.04	−0.06	0.83
family3	−0.07	0.02	0.05	0.02	0.00	**0.87**	−0.04	−0.09	0.04	0.02	0.04	0.04	−0.10	0.01	−0.04	0.08	0.74
family4	−0.03	0.00	−0.03	−0.02	0.06	**0.86**	0.00	0.08	−0.01	−0.05	0.00	−0.04	0.05	0.02	0.05	0.09	0.78
family1	0.12	−0.02	0.02	0.01	0.00	**0.79**	0.02	0.01	−0.03	−0.01	−0.08	−0.07	0.02	0.04	−0.07	−0.04	0.73
active	0.02	0.00	0.05	0.03	−0.06	−0.06	**0.93**	−0.06	0.05	−0.01	−0.01	0.00	0.03	−0.02	0.00	−0.02	0.85
excited	−0.04	−0.06	−0.05	0.02	0.03	0.01	**0.93**	0.03	0.02	−0.02	0.02	0.00	0.00	−0.01	0.02	0.04	0.80
happy	0.01	0.01	0.01	0.02	−0.03	−0.01	**0.92**	0.08	−0.05	0.01	0.01	−0.02	−0.03	0.02	0.00	0.02	0.85
enthuse.	−0.03	−0.05	0.01	−0.01	−0.01	−0.01	**0.90**	0.00	−0.01	−0.01	−0.01	−0.01	0.07	0.06	0.00	0.04	0.84
delighted	0.07	0.03	−0.02	0.01	−0.02	0.03	**0.88**	−0.02	0.01	−0.07	−0.01	0.01	−0.02	−0.01	0.02	−0.02	0.80
annoyed	0.02	0.02	−0.04	−0.03	0.06	−0.01	0.04	**0.95**	−0.05	0.03	−0.03	0.02	−0.07	0.01	0.07	−0.04	0.81
resentful	−0.04	0.00	0.01	−0.01	0.03	0.06	−0.02	**0.83**	0.07	−0.02	0.02	0.05	0.02	−0.06	0.01	0.06	0.75
irritable	0.06	0.04	−0.04	0.00	−0.13	0.04	0.09	**0.83**	0.00	0.04	0.04	0.01	−0.01	0.03	−0.05	−0.03	0.70
indifferent	−0.07	−0.04	0.17	0.12	−0.05	−0.07	−0.18	**0.54**	0.05	−0.08	−0.02	0.00	−0.01	0.02	−0.12	0.02	0.51
nervous	0.08	0.07	0.01	−0.01	0.04	−0.02	0.01	−0.08	**0.92**	−0.03	0.03	0.05	−0.06	0.04	−0.02	0.02	0.73
afraid	−0.03	0.00	−0.02	−0.02	0.10	−0.04	0.08	0.06	**0.84**	0.06	−0.02	0.01	−0.04	−0.06	−0.01	0.06	0.72
sad	−0.09	0.00	0.03	0.01	−0.08	0.07	−0.06	0.05	**0.80**	0.01	−0.04	−0.06	0.05	0.03	0.09	−0.07	0.71
anxious	0.11	−0.17	−0.08	0.02	−0.06	0.00	−0.06	0.15	**0.49**	−0.06	0.04	−0.07	0.15	0.00	−0.04	−0.04	0.56
identity1	−0.02	−0.13	−0.03	0.04	0.02	0.03	−0.06	0.02	−0.04	**0.96**	0.03	0.02	0.00	0.04	−0.01	0.03	0.85
identity2	0.01	−0.13	0.02	0.00	0.01	−0.01	−0.03	0.02	−0.06	**0.96**	0.03	0.04	0.04	0.02	−0.01	0.00	0.87
identity3	0.03	0.01	0.01	0.01	−0.02	−0.02	0.05	0.03	0.03	**0.90**	0.01	−0.02	0.03	−0.05	−0.01	−0.03	0.81
identity4	−0.02	0.14	0.00	0.01	−0.06	0.05	0.01	−0.05	0.06	**0.82**	−0.04	0.01	−0.10	0.01	0.01	−0.02	0.73
identity5	−0.03	0.23	−0.02	−0.07	−0.01	−0.03	−0.07	−0.01	0.03	**0.74**	−0.03	−0.08	0.05	−0.03	0.05	0.03	0.71
materi.2	−0.02	0.00	−0.02	0.06	0.01	0.05	−0.02	0.01	0.01	−0.02	**0.87**	0.03	0.02	0.03	−0.05	0.05	0.74
materi.1	0.00	−0.01	−0.04	0.01	0.01	0.04	0.03	−0.02	−0.06	0.11	**0.86**	−0.01	−0.04	0.01	−0.06	0.04	0.74
materi.3	0.05	0.01	−0.02	−0.03	0.00	−0.08	−0.03	−0.02	0.03	−0.08	**0.81**	−0.03	0.01	−0.02	0.05	0.01	0.71
materi.4	−0.01	0.06	0.10	−0.08	−0.06	−0.02	0.02	0.04	0.02	−0.02	**0.75**	−0.02	0.03	0.03	0.08	−0.15	0.60
pragm.2	0.03	−0.08	−0.02	0.07	0.09	−0.04	−0.10	0.06	−0.02	0.01	0.09	**0.84**	0.05	−0.02	0.01	−0.01	0.72
pragm.1	−0.02	0.02	0.04	−0.04	0.02	0.00	0.03	0.10	−0.01	0.00	0.01	**0.78**	0.08	0.04	−0.09	0.03	0.64
pragm.3	0.05	0.10	−0.01	−0.09	0.02	−0.01	0.06	0.01	0.04	−0.02	−0.12	**0.75**	0.01	0.03	−0.05	0.01	0.63
pragm.4	−0.04	0.01	0.01	0.04	−0.20	0.05	0.01	−0.11	−0.01	−0.02	−0.03	**0.62**	−0.11	−0.08	0.21	−0.02	0.43
respons.1	−0.02	−0.02	0.04	0.01	−0.12	0.02	0.06	−0.07	0.01	0.01	−0.01	0.02	**0.91**	−0.01	−0.06	0.02	0.80
respons.3	−0.05	0.06	−0.04	−0.03	0.10	0.01	0.01	0.05	−0.07	−0.11	0.06	0.02	**0.82**	−0.05	0.05	0.08	0.75
respons.2	−0.01	0.01	0.03	0.01	−0.02	−0.08	−0.01	−0.05	0.06	0.18	−0.04	0.00	**0.77**	0.01	0.00	0.00	0.73
altruistic1	0.01	0.02	0.00	0.00	0.00	−0.02	0.01	0.02	0.01	0.02	0.04	0.03	−0.09	**0.87**	0.11	−0.06	0.75
altruistic2	0.05	−0.11	0.03	0.07	−0.02	0.05	0.03	−0.09	0.06	0.02	0.03	0.00	−0.02	**0.85**	−0.05	−0.01	0.75
altruistic3	−0.10	0.00	−0.09	0.03	−0.01	0.00	−0.04	−0.03	0.00	−0.04	0.04	0.01	0.06	**0.84**	0.09	0.01	0.71
altruistic4	−0.05	0.06	0.03	−0.05	0.07	0.01	0.02	0.14	−0.07	−0.03	−0.05	−0.09	−0.02	**0.81**	0.02	0.04	0.67
altruistic5	0.05	0.08	0.08	−0.05	0.00	−0.04	0.02	−0.04	0.03	0.05	−0.02	0.01	0.00	**0.74**	−0.16	0.10	0.65
resolve3	0.05	−0.04	0.02	0.02	0.06	−0.06	0.04	−0.05	0.03	0.04	0.04	0.04	−0.17	−0.04	**0.84**	0.05	0.73
resolve2	−0.03	0.00	0.05	−0.02	−0.03	0.04	0.12	0.03	−0.05	−0.04	−0.02	0.04	0.01	0.06	**0.76**	−0.02	0.73
resolve4	−0.09	0.00	0.13	0.02	0.04	0.05	0.03	0.00	0.08	0.04	0.08	−0.05	−0.06	−0.16	**0.71**	0.16	0.65
resolve5	0.11	0.01	−0.12	0.07	−0.06	0.00	−0.16	−0.08	0.01	−0.09	−0.06	0.03	0.15	0.14	**0.69**	−0.02	0.60
resolve1	−0.03	0.10	0.05	−0.06	−0.01	−0.02	0.01	0.11	−0.04	0.06	−0.05	−0.03	0.13	0.09	**0.68**	−0.09	0.63
public1	−0.08	0.15	0.05	−0.03	−0.13	0.07	0.06	−0.08	0.01	0.01	−0.01	0.02	−0.02	0.01	−0.10	**0.86**	0.74
public2	−0.10	0.07	0.04	0.01	−0.01	0.05	0.04	−0.09	0.04	−0.04	0.02	0.02	0.01	0.03	−0.03	**0.82**	0.72
public3	−0.05	0.06	−0.02	0.00	0.12	−0.01	0.00	0.18	−0.04	−0.05	−0.04	−0.03	0.02	−0.04	0.08	**0.72**	0.60
public4	0.25	−0.21	−0.09	0.00	0.05	−0.03	−0.03	−0.02	−0.04	−0.01	0.01	−0.07	0.15	0.01	0.12	**0.61**	0.64
public5	0.14	−0.15	−0.03	0.00	0.02	−0.06	−0.03	0.03	0.02	0.16	0.00	0.07	0.01	0.10	0.11	**0.60**	0.59

Bold values indicate the primary factor loading of each item on its corresponding factor.

*Social Cognition*: Four items were removed due to high cross-loadings (difference < 0.20), and 14 items were removed for brevity. The final 6 factors were labeled Social Support (explaining 5.70% of variance), Social Trust (3.42%), Social Efficacy (4.62%), Social Safety (3.75%), Social Satisfaction (2.56%), and Family Support (2.23%).

*Social Emotion*: A 3-factor solution was extracted. Five items were excluded due to cross-loadings, and four items were removed for brevity. The factors were labeled Positive Emotion (6.39%), Oppositional Emotion (2.08%), and Vulnerable Emotion (1.68%).

*Social Values*: Eight items were removed (two due to low communalities <0.30, three due to cross-loadings, three due to brevity). The factors were labeled National Identity (25.84%), Materialism (1.85%), Pragmatism (1.57%), and Social Responsibility (1.44%).

*Social Behavioral Tendencies*: Four items were removed due to cross-loadings, and two items were removed for brevity. The factors were labeled Altruistic Behavior (3.97%), Conflict Resolution (1.96%) and Public Participation (2.81%).

### 3.3 Confirmatory factor analysis (CFA results on sample 2)

Due to moderate inter-factor correlations, the structure matrix revealed substantial cross-loadings, consistent with known limitations of higher-order EFA (Gorsuch, 1983). We therefore relied on CFA for higher-order structural validation, as recommended for theory-driven models (Brown, 2015; Kline, 2016).

Confirmatory Factor Analysis (CFA) was conducted on Sample 2 (*n* = 657) using AMOS 24.0 with Maximum Likelihood estimation. The factor structures derived from the EFA were tested for each of the four subscales. Model fit was evaluated based on the following criteria: $\chi^{2}$ /d*f* < 5, CFI ≥ 0.90, TLI ≥ 0.90, RMSEA ≤ 0.08, and SRMR ≤ 0.08 (Hu and Bentler, 1999).

We initially employed 73 items as observed variables to construct a model comprising 16 first-order factors and 4s-order factors in a confirmatory factor analysis (CFA). The results showed that the factor loading of materialism on its corresponding social value factor was non-significant (*β* = 0.02, *p* = 0.564). Accordingly, based on the CFA results, the four items measuring materialism were removed from the scale. This yielded a final scale with 69 items, 15 first-order factors, and 4s-order factors.

The specification of a third-order Social Mentality factor is theoretically grounded in the proposition that social cognition, social emotion, social values, and social behavioral tendencies are not merely correlated domains but are systematically organized components of a single overarching construct—the shared psychological response of individuals to the macro-social environment (Ma, 2008; Wang, 2023). In other words, these four components are hypothesized to covary because they are jointly driven by a common latent disposition, namely social mentality. Following the conceptualization, specification, and validation framework for higher-order constructs outlined by Kim and Byon (2021), we therefore modeled social mentality as a third-order factor that captures the common variance shared among the four second-order components and accounts for their covariation.

**Figure 1:**
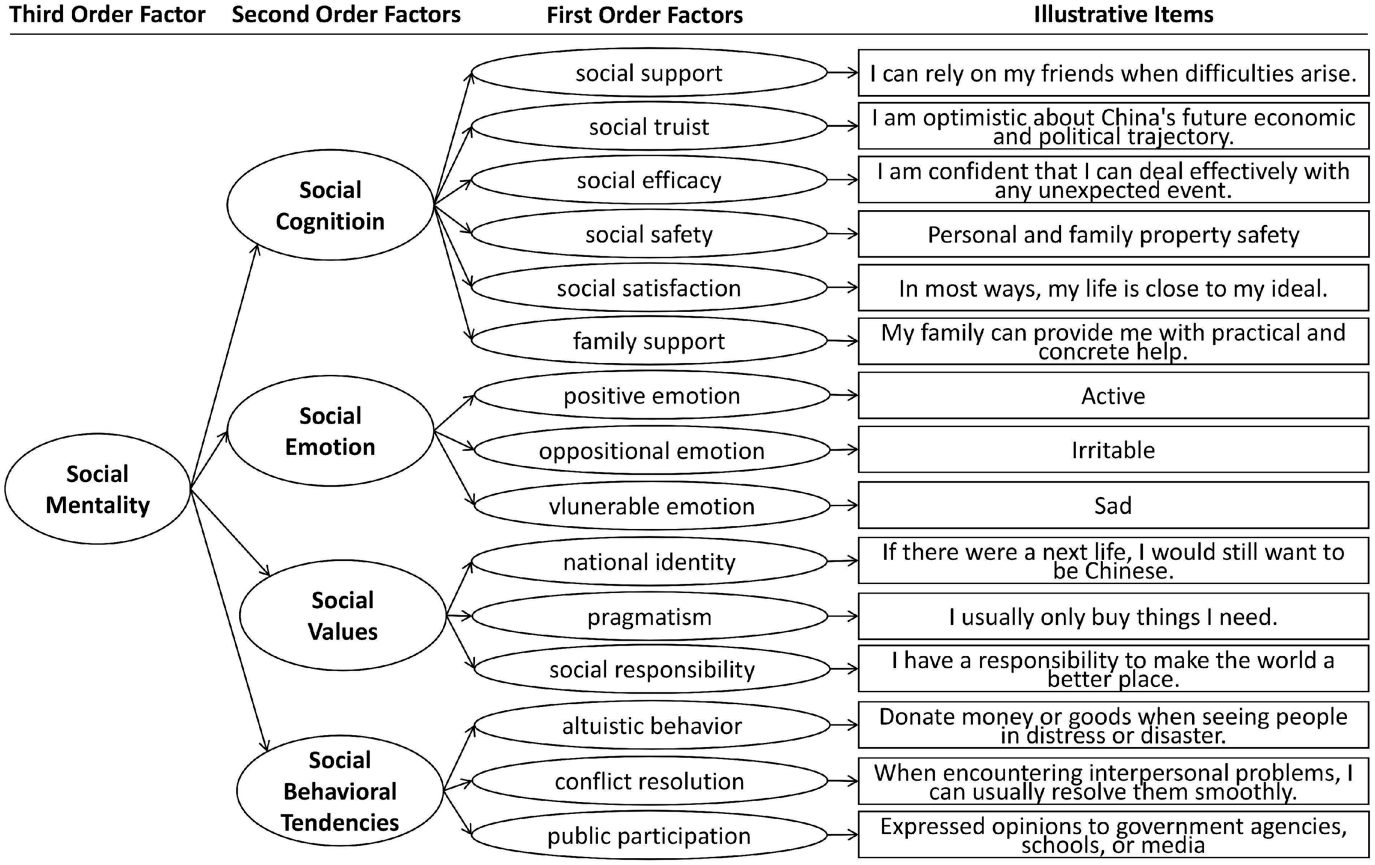
The higher order structural illustration for the CUSSMQ.

Subsequently, using the 69 items as observed variables, we built a model with 15 first-order factors and 4s-order factors. We then compared three alternative specifications of the relationships among the four second-order factors: a correlated model, a bifactor model, and a higher-order factor model (i.e., all second-order factors loading onto a single higher-order factor). The fit indices for these models are presented in Table 3. Only the higher-order factor model (all paths *p* < 0.001) exhibited acceptable fit indices. Moreover, the higher-order factor model fit the data significantly better than the bifactor model (Δ*χ*^2^ = 146.11, Δd*f* = 6, *p* < 0.001) and the four-factor correlated model (Δ*χ*^2^ = 1080.55, Δd*f* = 2, *p* < 0.001). Following the illustration by Kim and Byon (2021). The final higher-order factor structure of the CUSSMQ is illustrated in Figure 1.

**Table 3:** Fit indices for different models for the CUSSMQ (sample 2).

Scale structure	$\chi^{2}$ /d*f*	SRMR	RMSEA [90% CI]	TLI	CFI
Four-factor correlated model	2.38	0.516	0.06 [0.044, 0.047]	0.869	0.874
Bifactor model	1.97	0.244	0.038 [0.037, 0.040]	0.909	0.913
Higher order factor model	1.90	0.069	0.037 [0.035, 0.039]	0.914	0.918

### 3.4 Reliability and construct validity

Reliability Internal consistency and split-half reliability were assessed to evaluate the stability of the scale. As shown in Table 4, Cronbach’s *α* coefficients for the four subscales ranged from 0.73 to 0.95 (Social Cognition: 0.88–0.91; Social Emotion: 0.81–0.95; Social Values: 0.73–0.92; Social Behavioral Tendencies: 0.84–0.88). Additionally, split-half reliability coefficients ranged from 0.74 to 0.91, indicating excellent reliability across all dimensions.

**Table 4:** Reliability and convergent validity for lower order factors.

Subscale/dimension	No. of items	Cronbach’s *α*	Split-half	CR	AVE
Social cognition	29	0.88–0.91	0.79–0.88		
Social support	5	0.91	0.88	0.93	0.73
Social trust	5	0.90	0.84	0.92	0.74
Social efficacy	5	0.90	0.81	0.93	0.73
Social safety	5	0.88	0.81	0.91	0.67
Social satisfaction	5	0.89	0.79	0.92	0.69
Family support	4	0.89	0.87	0.92	0.75
Social emotion	13	0.81–0.95	0.79–0.91		
Positive emotion	5	0.95	0.91	0.96	0.83
Oppositional emotion	4	0.82	0.80	0.89	0.66
Vulnerable emotion	4	0.81	0.79	0.88	0.64
Social values	12	0.73–0.92	0.74–0.83		
National identity	5	0.92	0.83	0.95	0.78
Pragmatism	4	0.73	0.74	0.84	0.57
Social responsibility	3	0.83	0.76	0.90	0.75
Social behavior tendencies	15	0.84–0.88	0.76–0.83		
Altruistic behavior	5	0.88	0.83	0.91	0.68
Conflict resolution	5	0.84	0.81	0.88	0.65
Public participation	5	0.84	0.76	0.89	0.61

Convergent Validity Convergent validity was evaluated using Composite Reliability (CR) and Average Variance Extracted (AVE) based on the CFA factor loadings. As presented in Table 4, all CR values exceeded the recommended threshold of 0.60, and AVE values were all above 0.50 (Fornell and Larcker, 1981). These results indicate satisfactory convergent validity for the measurement model (see Table 4).

For the higher-order factors, standardized loadings, composite reliability (CR), and average variance extracted (AVE) are presented in Table 5. All factor loadings from the lower-order factors to their respective second-order factors, as well as from the second-order factors to the third-order factor, were statistically significant (all *p* < 0.001). It should be noted that in a hierarchical reflective model, the loading of a second-order factor on the third-order factor represents the relationship between the estimated latent composite score of that second-order factor (which is a weighted combination of its first-order dimensions) and the third-order construct. Therefore, a relatively strong third-order loading (e.g., 0.77 for Social Values) does not require every first-order dimension to have a high loading (e.g., 0.35 for Pragmatism).

**Table 5:** Standardized factor loadings and convergent validity for higher order factors.

Higher-order factor	Lower-order factor	Standardized loading	CR	AVE
Second order: social cognition	Social support	0.59^***^	0.83	0.46
	Social trust	0.73^***^		
	Social efficacy	0.71^***^		
	Social safety	0.57^***^		
	Social satisfaction	0.80^***^		
	Family support	0.62^***^		
Social order factor: social emotion	Positive emotion	0.80^***^	0.63	0.37
	Oppositional emotion	−0.42^***^		
	Vulnerable emotion	−0.55^***^		
Second order factor: social values	National identity	0.63^***^	0.66	0.42
	Pragmatism	0.35^***^		
	Social responsibility	0.86^***^		
Second order factor: social behavioral tendencies	Altruistic behavior	0.63^***^	0.75	0.50
	Public participation	0.84^***^		
	Conflict resolution	0.64^***^		
Third order factor: social mentality	Social cognition	0.96^***^	0.93	0.77
	Social emotion	0.94^***^		
	Social values	0.77^***^		
	Social behavioral tendencies	0.83^***^		

***p < 0.001.

For the second-order factors, CR values ranged from 0.63 to 0.83, all exceeding the threshold of 0.60 (Fornell and Larcker, 1981). Although the AVE for some second-order factors (e.g., Social Support: AVE = 0.46; Social Emotion: AVE = 0.37; Social Values: AVE = 0.42) fell slightly below the conventional criterion of 0.50, this is not uncommon in higher-order measurement models. As noted by Hair et al. (2010), if CR is adequate, convergent validity can still be considered sufficient even when AVE is lower than 0.50, especially when the lower-order factors themselves demonstrate strong reliability and convergent validity (see Table 4). For the third-order factor (Social Mentality), CR was 0.93 and AVE was 0.77.

Discriminant Validity Discriminant validity was examined using the Heterotrait-Monotrait Ratio (HTMT). As shown in Table 6, the largest HTMT values between each factors was 0.70 < 0.85 (Henseler et al., 2015). This confirms that each dimension of the CUSSMQ represents a distinct construct.

**Table 6:** Discriminant validity analysis (Heterotrait-Monotrait ratio).

Dimension	1	2	3	4	5	6	7	8	9	10	11	12	13	14	15
1. Social support	1.00														
2. Social trust	0.34	1.00													
3. Social efficacy	0.37	0.42	1.00												
4. Social safety	0.31	0.53	0.39	1.00											
5. Social satisfaction	0.40	0.57	0.58	0.45	1.00										
6. Family support	0.56	0.33	0.42	0.31	0.50	1.00									
7. Positive emotion	0.42	0.48	0.49	0.38	0.64	0.42	1.00								
8. Oppositional emotion	0.27	0.28	0.32	0.26	0.39	0.30	0.32	1.00							
9. Vulnerable emotion	0.27	0.29	0.42	0.30	0.48	0.34	0.37	0.70	1.00						
10. National identity	0.27	0.62	0.22	0.34	0.29	0.23	0.33	0.20	0.15	1.00					
11. Pragmatism	0.04	0.13	0.23	0.24	0.22	0.11	0.10	0.15	0.13	0.18	1.00				
12. Social responsibility	0.39	0.51	0.40	0.32	0.39	0.29	0.46	0.18	0.17	0.53	0.27	1.00			
13. Altruistic behavior	0.39	0.34	0.30	0.25	0.25	0.28	0.32	0.22	0.13	0.39	0.18	0.56	1.00		
14. Conflict resolution	0.49	0.36	0.61	0.35	0.45	0.36	0.53	0.28	0.30	0.27	0.26	0.55	0.50	1.00	
15. Public participation	0.35	0.25	0.42	0.13	0.32	0.24	0.46	0.10	0.12	0.26	0.17	0.56	0.45	0.62	1.00

### 3.5 Criterion-related validity

To assess criterion-related validity, the relationships between the CUSSMQ and the Prosocial Tendencies Measure (PTM) were examined. As hypothesized, the CUSSMQ demonstrated robust correlations with prosocial behaviors in theoretical expected directions.

As presented in Table 7, the total scores of the Social Cognition, Social Values, and Social Behavioral Tendencies subscales, as well as their positive dimensions (e.g., Social Responsibility, Altruistic Behavior), showed significant positive correlations with all dimensions of prosocial tendencies (*r*s ranged from 0.08 to 0.67, *p* < 0.05). Crucially, the scale effectively differentiated between adaptive and maladaptive mentality. The Oppositional Emotion and Vulnerable Emotion factors exhibited significant negative correlations with prosocial tendencies (*r*s ranged from −0.21 to −0.09, *p* < 0.05). Overall, these results provide preliminary evidence for the criterion-related validity of the CUSSMQ, though the scope of criterion coverage remains limited.

**Table 7:** Correlations between CUSSMQ dimensions and prosocial tendencies.

Subscale/dimension	Prosocial behavior	Public	Anonymous	Altruistic	Compliant	Emotional	Emergency
Social cognition subscale	0.39^***^	0.33^***^	0.27^***^	0.34^***^	0.33^***^	0.33^***^	0.34^***^
Social support	0.33^***^	0.29^***^	0.19^***^	0.30^***^	0.26^***^	0.31^***^	0.33^***^
Social trust	0.31^***^	0.24^***^	0.22^***^	0.26^***^	0.30^***^	0.30^***^	0.24^***^
Social efficacy	0.28^***^	0.25^***^	0.21^***^	0.27^***^	0.23^***^	0.23^***^	0.25^***^
Social safety	0.30^***^	0.26^***^	0.20^***^	0.28^***^	0.25^***^	0.24^***^	0.28^***^
Social satisfaction	0.28^***^	0.24^***^	0.23^***^	0.23^***^	0.23^***^	0.23^***^	0.20^***^
Family support	0.20^***^	0.17^***^	0.15^***^	0.17^***^	0.18^***^	0.15^***^	0.21^***^
Social emotion subscale	0.28^***^	0.25^***^	0.20^***^	0.27^***^	0.23^***^	0.24^***^	0.24^***^
Positive emotion	0.34^***^	0.34^***^	0.21^***^	0.28^***^	0.27^***^	0.31^***^	0.28^***^
Oppositional emotion	−0.18^***^	−0.11^***^	−0.13^***^	−0.21^***^	−0.15^***^	−0.15^***^	−0.16^***^
Vulnerable emotion	−0.12^***^	−0.10^***^	−0.10^***^	−0.13^***^	−0.09^***^	−0.09^***^	−0.09^***^
Social values subscale	0.24^***^	0.26^***^	0.08^**^	0.16^***^	0.23^***^	0.25^***^	0.22^***^
National identity	0.32^***^	0.21^***^	0.22^***^	0.31^***^	0.29^***^	0.31^***^	0.27^***^
Pragmatism	0.25^***^	0.14^***^	0.24^***^	0.24^***^	0.22^***^	0.19^***^	0.20^***^
Social responsibility	0.54^***^	0.44^***^	0.38^***^	0.46^***^	0.46^***^	0.49^***^	0.48^***^
Social behavioral tendencies subscale	0.67^***^	0.57^***^	0.47^***^	0.58^***^	0.53^***^	0.60^***^	0.59^***^
Altruistic behavior	0.66^***^	0.44^***^	0.48^***^	0.64^***^	0.54^***^	0.59^***^	0.64^***^
Conflict resolution	0.52^***^	0.49^***^	0.34^***^	0.42^***^	0.42^***^	0.48^***^	0.44^***^
Public participation	0.46^***^	0.45^***^	0.33^***^	0.39^***^	0.34^***^	0.41^***^	0.39^***^

***p < 0.001.

### 3.6 Nomological validity

**Figure 2:**
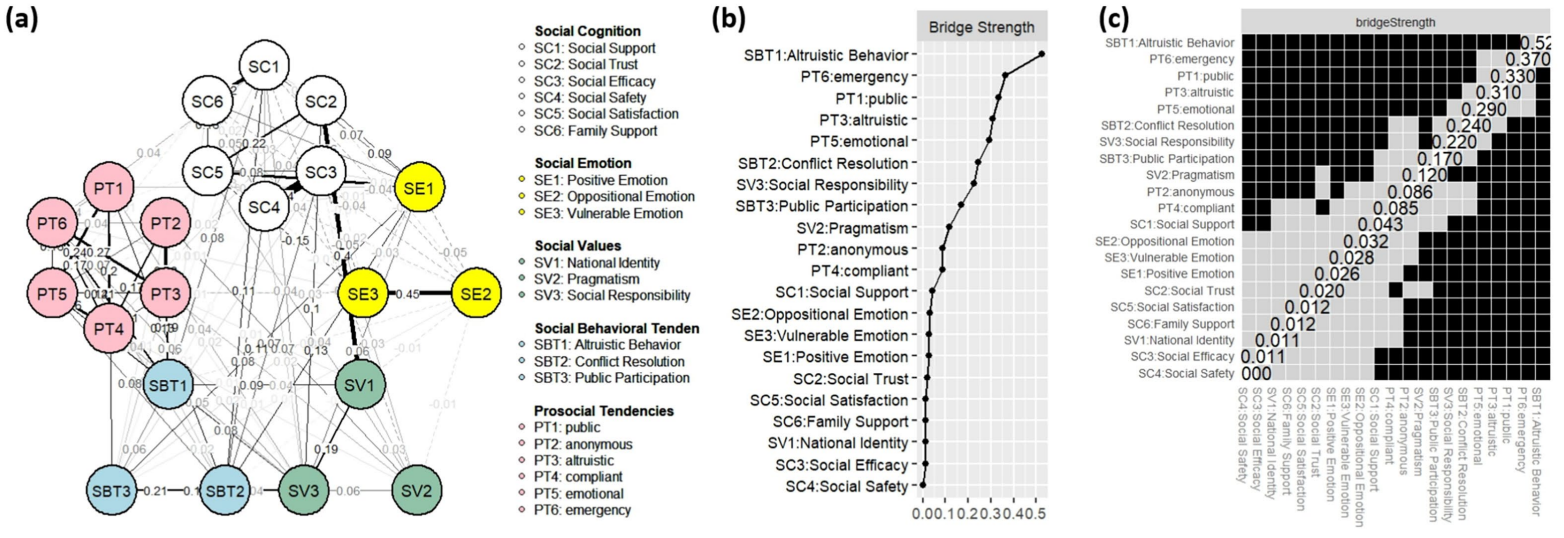
**(a)** Network graph; **(b)** Nodes’ bridge strength in order; **(c)** Bridge strength comparison between nodes. Note. Black cells means that two nodes showed significant difference on bridge strength.

To establish the nomological validity of the developed instrument, we conducted a network analysis and examined bridge strength between social mentalities and prosocial tendencies. The network, as shown in Figure 2a, demonstrated good stability (CS for node strength, bridge strength, and edges = 0.89, 0.89, and 0.89, respectively), with a density of 0.04.

Bridge strength quantifies the degree to which a construct connects different theoretical communities in the network, thereby reflecting nomological validity. As presented in Figure 2b, when bridging with prosocial tendencies, constructs belonging to social behavioral tendencies exhibited the highest bridge strength values (ranging from 0.17 to 0.52), followed by social values (ranging from 0.01 to 0.22). In contrast, constructs reflecting social cognition (ranging from 0.00 to 0.04) and social emotions (all 0.03) showed substantially lower bridge strength.

Pairwise significance comparisons (see Figure 2c) further confirmed that the bridge strengths of nodes from social behavioral tendencies and social values were mostly significantly higher than those of social cognition or social emotion (*p* < 0.05 for all relevant comparisons indicated by black cells).

## 4 Discussion

### 4.1 Summary of findings

The present study successfully developed and validated the Chinese University Students’ Social Mentality Questionnaire (CUSSMQ) based on the Cognition-Emotion-Values-Behavior four-component model (Ma, 2008; Wang, 2023).

The results confirmed a stable 15-factor structure across the four subscales, highlighting culturally specific dimensions such as the separation of Family Support from general Social Support. Psychometric evaluations demonstrated that the CUSSMQ possesses excellent internal consistency, construct validity, and discriminant validity, meeting rigorous psychometric standards. Furthermore, criterion-related validity analysis revealed that the positive mentality dimensions of the CUSSMQ significantly predict prosocial behaviors. These findings suggest that the CUSSMQ is a reliable and sensitive instrument for capturing the multifaceted nature of social mentality among contemporary Chinese university students.

Our findings confirm that social mentality is not a monolithic construct but a multidimensional system. Consistent with Yang (2006) and Wang (2023), who defined social mentality as the “psychological mirror” of social changes, the CUSSMQ successfully captures the complexity of contemporary Chinese students’ psychology. Specifically, the identification of culturally specific dimensions—such as the separation of Family Support from general Social Support within cognition and the differentiation of distinct emotional subtypes—demonstrates that the scale is sensitive to the unique micro-ecology of Chinese universities (Bronfenbrenner, 1979).

### 4.2 Interpretation of the factor structure: cultural and contextual validity

*The Indigenization of Social Cognition*: Unlike Western models that often lump social support into a single construct, our study separated Family Support as a distinct factor. This aligns with Xin and Su (2020), who noted in their 50-year review that despite rising individualism, familial ties remain a core buffer for Chinese people. Furthermore, the inclusion of Social Safety and Social Satisfaction echoes Fong and Zhong (2021), who emphasized that a sense of gain and security are foundational to students’ happiness.

*Capturing the Anxiety of the Digital Age*: In the Social Emotion subscale, we identified Vulnerable Emotion (e.g., anxiety, fragility). This resonates with recent findings by Wang and Zhang (2024) and Gong et al. (2025), which highlight the prevalence of social anxiety and Fear of Missing Out (FoMO) among Chinese youth in the digital era. The CUSSMQ effectively operationalizes these diffuse anxieties into a measurable construct.

*Decoding Lying Flat vs. Involution*: Our identification of Pragmatism as a distinct value orientation offers a theoretical lens to understand the Lying Flat phenomenon. He et al. (2025) suggested that low self-esteem drives some youth to lie flat (a behavioral withdrawal). Our findings extend this by suggesting that Pragmatism (focusing on utility) may be an adaptive response to Cui et al.’s (2023) concept of resource scarcity, reflecting a rational reallocation of effort under intense competitive pressure rather than mere withdrawal.

### 4.3 Discussion of key findings

A noteworthy finding of this study is that the CUSSMQ effectively differentiated between adaptive and maladaptive mentality in relation to prosocial behavior. While the positive mentality dimensions (e.g., Social Trust, Social Responsibility, and Altruistic Behavior) showed significant positive correlations with prosocial tendencies, the negative emotional dimensions (Oppositional Emotion and Vulnerable Emotion) were negatively associated with them.

This pattern supports the theoretical expectation that an individual’s affective and cognitive orientation toward the social environment shapes their propensity for prosocial engagement. Consistent with the mechanism proposed by Miao et al. (2021), in which positive emotional and cognitive resources facilitate altruism, our results indicate that students with a more adaptive social mentality are more inclined toward prosocial action, whereas those characterized by hostility or vulnerability are less so. This nuance demonstrates that the CUSSMQ has high discriminant validity in distinguishing different motivational pathways underlying social behavior.

### 4.4 Practical implications

The development of the CUSSMQ offers meaningful implications for higher education management and psychological counseling practices.

First, Establish a Dynamic Monitoring Mechanism. Social mentality is highly context-dependent. Zhao et al. (2022) demonstrated through a longitudinal study that university students’ mentality fluctuates significantly during public crises. Universities can use the CUSSMQ as a routine screening tool to track the dynamic trajectory of students’ cognition and emotions, enabling early identification of potential risks.

Second, Implement “Precision Intervention” based on Emotional Subtypes. The CUSSMQ’s distinction between Oppositional and Vulnerable emotions allows for stratified interventions. For students scoring high on Vulnerable Emotion (e.g., anxiety, fragility), interventions should focus on enhancing social support and reducing the “relative deprivation” caused by social comparison. Conversely, for those exhibiting high Oppositional Emotion, educational programs should prioritize conflict resolution skills and rational expression channels to mitigate hostility.

Third, Cultivate Adaptive Value Orientations. Our findings suggest that Pragmatism represents a rational adaptation to competitive pressure. Consistent with Yu and Liu (2014), educators should help students channel competitive pressures constructively. Value education. Value education should aim to transform the anxiety stemming from resource scarcity (Cui et al., 2023) into constructive motivation, encouraging students to pursue self-actualization rather than mere material accumulation.

### 4.5 Limitations and future directions

Several limitations of this study should be acknowledged. First, the cross-sectional design limits our ability to make causal inferences between social mentality and prosocial behaviors. Future research should adopt longitudinal designs, similar to Lam et al. (2020), to explore how social mentality evolves over the course of university life.

Second, the current criterion-related validity evidence should be considered preliminary rather than comprehensive. The present study relied solely on the Prosocial Tendencies Measure (PTM) as the criterion, which presents two limitations. On the one hand, prosocial behavioral tendencies are conceptually overlapping with the Social Behavioral Tendencies subscale of the CUSSMQ, introducing a risk of criterion contamination and circular reasoning. On the other hand, the PTM primarily taps into the behavioral domain, leaving the Social Cognition and Social Emotion components without independent criterion validation. Future research should employ a broader range of theoretically relevant criteria to establish a more complete nomological network for the CUSSMQ. Specifically, emotional criteria (e.g., anxiety scales, the Positive and Negative Affect Schedule; Watson et al., 1988), cognitive criteria (e.g., items from the World Values Survey, cognitive flexibility measures), and non-self-report criteria (e.g., behavioral experiments, third-party ratings, macro-social indicators such as civic engagement records) should be incorporated to comprehensively test the predictive and convergent validity of each subscale. In addition, the nomological validity evidence presented in this study (Section 3.6) was limited to examining the CUSSMQ’s network relationships with a single external criterion (prosocial tendencies). Future research should establish a more comprehensive nomological network by examining theoretically predicted relationships with constructs such as subjective well-being (e.g., Diener et al., 1985), social alienation (e.g., Dean, 1961), political efficacy (e.g., Niemi et al., 1991), and generalized trust (e.g., Yamagishi and Yamagishi, 1994).

Third, data were collected exclusively via self-report measures. Although common method bias was not found to be a serious issue, future studies could incorporate implicit measures (Greenwald et al., 1998) or behavioral experiments to validate the explicit attitudes measured by the CUSSMQ.

Fourth, participants were recruited through convenience sampling via an online platform, which may introduce self-selection bias and limit the representativeness of the sample. Although the 10 participating universities were selected to cover different regions and institutional types, the voluntary nature of online recruitment may have overrepresented students with higher digital engagement or stronger interest in the research topic. Future validation studies should adopt stratified random sampling methods across systematically selected regions, institution types, and academic disciplines to enhance the generalizability of the CUSSMQ norms.

Fifth, it should be noted that a few second-order dimensions exhibited standardized loadings below the conventional threshold of 0.50. The negative loadings of Oppositional Emotion (−0.42) and Vulnerable Emotion (−0.55) on the Social Emotion factor are theoretically expected rather than problematic: consistent with the relative independence of positive and negative affect (Watson and Tellegen, 1985), higher overall Social Emotion is associated with lower negative emotional orientations. Pragmatism (0.35) was retained because it captures a culturally meaningful facet of Chinese university students’ social values. Although these modest loadings may marginally affect the convergent validity of the corresponding constructs, future studies may consider item refinement or further validation of these dimensions.

Furthermore, the present study measured social mentality at the individual level. Future research should collect data from multiple distinct groups (e.g., different universities or regions) and employ aggregation indices (e.g., r_wg, ICC; James et al., 1984; Bliese, 2000) to empirically verify within-group consensus and validate the inference from individual measurement to group-level social mentality.

## 5 Conclusion

This study successfully developed and validated the Chinese University Students’ Social Mentality Questionnaire (CUSSMQ). The scale features a stable four-component structure (Cognition, Emotion, Values, Behavior) comprising 15 factors, with robust psychometric properties. By capturing culturally specific dimensions such as Family Support and the separation of Family Support from general Social Support and the differentiation of distinct emotional subtypes, the CUSSMQ provides a sensitive and reliable instrument for researchers and educators. It serves as a valuable tool for understanding the micro-ecology of contemporary Chinese university students and for designing targeted psychological interventions.

## Data Availability

The original contributions presented in the study are included in the article/Supplementary material, further inquiries can be directed to the corresponding author.
