# Supplementary material for: Development and validation of the Chinese university students’ social mentality questionnaire: a four-component model study
Source: Front Psychol. 2026 Jun 24;17:1804407. doi: 10.3389/fpsyg.2026.1804407 (PMC13341851; doi:10.3389/fpsyg.2026.1804407)
Supplement: Supplementary file 1 [file Supplementary_file_1.DOCX]

**Appendix A**

**Chinese University Students' Social Mentality Questionnaire (CUSSMQ)**

中国大学生社会心态调查问卷

*69 Items, 15 Dimensions, 4 Subscales*

**Note:** All items are rated on a 5-point Likert scale. The specific anchors vary by dimension as indicated in the instructions for each section. Negative items (Oppositional Emotion and Vulnerable Emotion) are reverse-coded so that higher scores reflect more positive or adaptive social mentality. The Materialism dimension, initially identified in the EFA, was removed following confirmatory factor analysis due to a non-significant higher-order loading; the final scale therefore comprises 69 items across 15 first-order dimensions. The Social Satisfaction items were adapted from the Satisfaction with Life Scale (Diener et al., 1985).

**Subscale I: Social Cognition (社会认知)**

**Social Support** (社会支持感)

*To what extent do you agree with the following statements? (1 = Strongly Disagree, 5 = Strongly Agree)*

| **Item** | **1** | **2** | **3** | **4** | **5** |
| --- | --- | --- | --- | --- | --- |
| 在发生困难时我可以依靠我的朋友们。 *I can rely on my friends when difficulties arise.* | ○ | ○ | ○ | ○ | ○ |
| 我能与朋友们讨论自己遇到的困难。 *I can discuss my difficulties with my friends.* | ○ | ○ | ○ | ○ | ○ |
| 我能够与有些人共享快乐与忧伤。 *I can share joys and sorrows with some people.* | ○ | ○ | ○ | ○ | ○ |
| 我的朋友们能与我分享快乐与忧伤。 *My friends can share their joys and sorrows with me.* | ○ | ○ | ○ | ○ | ○ |
| 在我遇到问题时有些人会出现在我的身旁。 *Some people are there for me when I have problems.* | ○ | ○ | ○ | ○ | ○ |

**Social Trust** (社会信任感)

*To what extent do you agree with the following statements? (1 = Strongly Disagree, 5 = Strongly Agree)*

| **Item** | **1** | **2** | **3** | **4** | **5** |
| --- | --- | --- | --- | --- | --- |
| 我对中国未来的经济和政治走势持乐观态度。 *I am optimistic about China's future economic and political trajectory.* | ○ | ○ | ○ | ○ | ○ |
| 社会环境为自己实现梦想提供了良好的条件。 *The social environment provides favorable conditions for me to realize my dreams.* | ○ | ○ | ○ | ○ | ○ |
| 现在只要个人足够努力，仍然有较大的机会在工作中出人头地、获得成功。 *As long as one works hard enough, there are still good opportunities for career success.* | ○ | ○ | ○ | ○ | ○ |
| 来自农村或城市贫困家庭的孩子仍然有较多机会通过个人努力考上重点大学。 *Children from rural or urban impoverished families still have many opportunities to enter top universities through personal effort.* | ○ | ○ | ○ | ○ | ○ |
| 总体上来说，我觉得当今社会是公平的。 *Overall, I feel that today's society is fair.* | ○ | ○ | ○ | ○ | ○ |

**Social Efficacy** (社会效能感)

*To what extent do you agree with the following statements? (1 = Strongly Disagree, 5 = Strongly Agree)*

| **Item** | **1** | **2** | **3** | **4** | **5** |
| --- | --- | --- | --- | --- | --- |
| 我自信能有效地应对任何突如其来的事情。 *I am confident that I can deal effectively with any unexpected event.* | ○ | ○ | ○ | ○ | ○ |
| 我能冷静的面对困难，因为我可以信赖自己处理问题的能力。 *I can remain calm when facing difficulties because I can rely on my own problem-solving abilities.* | ○ | ○ | ○ | ○ | ○ |
| 以我的才智，我定能应对意料之外的情况。 *With my resourcefulness, I can handle unforeseen situations.* | ○ | ○ | ○ | ○ | ○ |
| 无论什么事在我身上发生，我都能应付自如。 *No matter what happens to me, I can handle it with ease.* | ○ | ○ | ○ | ○ | ○ |
| 有麻烦的时候，我通常能想到一些应付的方法。 *When in trouble, I can usually think of ways to cope.* | ○ | ○ | ○ | ○ | ○ |

**Social Safety** (社会安全感)

*How safe do you feel in the following areas? (1 = Very Unsafe, 5 = Very Safe)*

| **Item** | **1** | **2** | **3** | **4** | **5** |
| --- | --- | --- | --- | --- | --- |
| 交通安全 *Traffic safety* | ○ | ○ | ○ | ○ | ○ |
| 医疗药品安全 *Medical and pharmaceutical safety* | ○ | ○ | ○ | ○ | ○ |
| 个人和家庭财产安全 *Personal and family property safety* | ○ | ○ | ○ | ○ | ○ |
| 人身安全 *Personal physical safety* | ○ | ○ | ○ | ○ | ○ |
| 总体上的社会安全状况 *Overall social safety conditions* | ○ | ○ | ○ | ○ | ○ |

**Social Satisfaction** (社会满意度)

*To what extent do you agree with the following statements? (1 = Strongly Disagree, 5 = Strongly Agree)*

| **Item** | **1** | **2** | **3** | **4** | **5** |
| --- | --- | --- | --- | --- | --- |
| 大多数情况下，我的生活接近理想状态。 *In most ways, my life is close to my ideal.* | ○ | ○ | ○ | ○ | ○ |
| 我的生活状态很好。 *The conditions of my life are excellent.* | ○ | ○ | ○ | ○ | ○ |
| 我对自己的生活感到满意。 *I am satisfied with my life.* | ○ | ○ | ○ | ○ | ○ |
| 到目前为止，我已经得到了我认为生活中最重要的事物。 *So far, I have gotten the important things I want in life.* | ○ | ○ | ○ | ○ | ○ |
| 即使生活从头再来我也没有什么想要改变的。 *If I could live my life over, I would change almost nothing.* | ○ | ○ | ○ | ○ | ○ |

**Family Support** (家庭支持感)

*To what extent do you agree with the following statements? (1 = Strongly Disagree, 5 = Strongly Agree)*

| **Item** | **1** | **2** | **3** | **4** | **5** |
| --- | --- | --- | --- | --- | --- |
| 我的家庭能够切实具体地给我帮助。 *My family can provide me with practical and concrete help.* | ○ | ○ | ○ | ○ | ○ |
| 在需要时我能够从家庭获得感情上的帮助和支持。 *I can obtain emotional help and support from my family when needed.* | ○ | ○ | ○ | ○ | ○ |
| 我能与自己的家庭谈论我遇到的困难。 *I can talk about my difficulties with my family.* | ○ | ○ | ○ | ○ | ○ |
| 我的家庭能心甘情愿协助我做出各种决定。 *My family is willing to help me make decisions.* | ○ | ○ | ○ | ○ | ○ |

**Subscale II: Social Emotion (社会情绪)**

**Positive Emotion** (积极情绪)

*Thinking about the past year, how often have you experienced the following emotions? (1 = Never, 5 = Always)*

| **Item** | **1** | **2** | **3** | **4** | **5** |
| --- | --- | --- | --- | --- | --- |
| 活跃的 *Active* | ○ | ○ | ○ | ○ | ○ |
| 充满热情的 *Enthusiastic* | ○ | ○ | ○ | ○ | ○ |
| 快乐的 *Happy* | ○ | ○ | ○ | ○ | ○ |
| 兴奋的 *Excited* | ○ | ○ | ○ | ○ | ○ |
| 欣喜的 *Delighted* | ○ | ○ | ○ | ○ | ○ |

**Oppositional Emotion** (对抗情绪)

*Thinking about the past year, how often have you experienced the following emotions? (1 = Never, 5 = Always)*

| **Item** | **1** | **2** | **3** | **4** | **5** |
| --- | --- | --- | --- | --- | --- |
| 易怒的 *Irritable* | ○ | ○ | ○ | ○ | ○ |
| 恼怒的 *Annoyed* | ○ | ○ | ○ | ○ | ○ |
| 怨恨的 *Resentful* | ○ | ○ | ○ | ○ | ○ |
| 冷漠的 *Indifferent* | ○ | ○ | ○ | ○ | ○ |

**Vulnerable Emotion** (脆弱情绪)

*Thinking about the past year, how often have you experienced the following emotions? (1 = Never, 5 = Always)*

| **Item** | **1** | **2** | **3** | **4** | **5** |
| --- | --- | --- | --- | --- | --- |
| 难过的 *Sad* | ○ | ○ | ○ | ○ | ○ |
| 害怕的 *Afraid* | ○ | ○ | ○ | ○ | ○ |
| 紧张的 *Nervous* | ○ | ○ | ○ | ○ | ○ |
| 焦虑的 *Anxious* | ○ | ○ | ○ | ○ | ○ |

**Subscale III: Social Values (社会价值观)**

**National Identity** (国家认同)

*To what extent do you agree with the following statements? (1 = Strongly Disagree, 5 = Strongly Agree)*

| **Item** | **1** | **2** | **3** | **4** | **5** |
| --- | --- | --- | --- | --- | --- |
| 大学生应牢固树立中国特色社会主义共同理想。 *University students should firmly uphold the common ideal of socialism with Chinese characteristics.* | ○ | ○ | ○ | ○ | ○ |
| 如果有下辈子，我还是愿意做中国人。 *If there were a next life, I would still want to be Chinese.* | ○ | ○ | ○ | ○ | ○ |
| 当两者发生冲突时，个人自由应该服务国家利益。 *When the two conflict, personal freedom should serve the national interest.* | ○ | ○ | ○ | ○ | ○ |
| 我对社会主义核心价值观的内容非常认同。 *I strongly identify with the content of the socialist core values.* | ○ | ○ | ○ | ○ | ○ |
| 大学生应该主动践行社会主义核心价值观。 *University students should actively practice the socialist core values.* | ○ | ○ | ○ | ○ | ○ |

**Pragmatism** (实用主义)

*To what extent do you agree with the following statements? (1 = Strongly Disagree, 5 = Strongly Agree)*

| **Item** | **1** | **2** | **3** | **4** | **5** |
| --- | --- | --- | --- | --- | --- |
| 在物质生活方面，我试图保持简单朴素。 *In terms of material life, I try to keep things simple and plain.* | ○ | ○ | ○ | ○ | ○ |
| 我通常只买我所需要的东西。 *I usually only buy things I need.* | ○ | ○ | ○ | ○ | ○ |
| 比起我认识的大多数人来说，我不那么重视物质的东西。 *Compared to most people I know, I place less emphasis on material things.* | ○ | ○ | ○ | ○ | ○ |
| 我喜欢花钱买一些不实用的东西。 *I enjoy spending money on things that are not practical.* | ○ | ○ | ○ | ○ | ○ |

**Social Responsibility** (社会责任)

*To what extent do you agree with the following statements? (1 = Strongly Disagree, 5 = Strongly Agree)*

| **Item** | **1** | **2** | **3** | **4** | **5** |
| --- | --- | --- | --- | --- | --- |
| 我有责任让世界变得更美好。 *I have a responsibility to make the world a better place.* | ○ | ○ | ○ | ○ | ○ |
| 我认为发生公共卫生、自然灾害、事故灾难等社会公共危机时大学生应该挺身而出。 *I believe university students should step forward during public crises such as public health emergencies, natural disasters, or accidents.* | ○ | ○ | ○ | ○ | ○ |
| 我做的事情对社会很重要。 *The things I do are important to society.* | ○ | ○ | ○ | ○ | ○ |

**Subscale IV: Social Behavioral Tendencies (社会行为倾向)**

**Altruistic Behavior** (利他行为)

*How willing would you be to do the following? (1 = Very Unwilling, 5 = Very Willing)*

| **Item** | **1** | **2** | **3** | **4** | **5** |
| --- | --- | --- | --- | --- | --- |
| 看到有人受困受灾而捐款捐物。 *Donate money or goods when seeing people in distress or disaster.* | ○ | ○ | ○ | ○ | ○ |
| 身边人有困难时主动帮助。 *Proactively help those around me when they are in difficulty.* | ○ | ○ | ○ | ○ | ○ |
| 当看到老人摔倒时给予帮助、救护。 *Help and assist when seeing an elderly person fall.* | ○ | ○ | ○ | ○ | ○ |
| 看到有人正在遭受欺诈或诈骗，善意提醒。 *Kindly warn someone who is being defrauded or scammed.* | ○ | ○ | ○ | ○ | ○ |
| 发现有人轻生倾向或行为，设法帮助。 *Try to help when noticing someone with suicidal tendencies or behavior.* | ○ | ○ | ○ | ○ | ○ |

**Conflict Resolution** (冲突解决)

*To what extent do you agree with the following statements? (1 = Strongly Disagree, 5 = Strongly Agree)*

| **Item** | **1** | **2** | **3** | **4** | **5** |
| --- | --- | --- | --- | --- | --- |
| 在语言不通的情况下，我仍能通过其他方式进行沟通。 *Even when there is a language barrier, I can still communicate through other means.* | ○ | ○ | ○ | ○ | ○ |
| 遇到人际关系问题，我往往能够顺利解决。 *When encountering interpersonal problems, I can usually resolve them smoothly.* | ○ | ○ | ○ | ○ | ○ |
| 与人交谈时，我能清楚地表达自己的观点。 *When talking with others, I can clearly express my viewpoints.* | ○ | ○ | ○ | ○ | ○ |
| 我有很好的演讲、辩论和展示的能力。 *I have good abilities in public speaking, debating, and presenting.* | ○ | ○ | ○ | ○ | ○ |
| 在沟通中，我能够理解别人的观点和意见。 *In communication, I can understand others' viewpoints and opinions.* | ○ | ○ | ○ | ○ | ○ |

**Public Participation** (公共参与)

*In the past year, how often have you done the following? (1 = Never, 5 = Always)*

| **Item** | **1** | **2** | **3** | **4** | **5** |
| --- | --- | --- | --- | --- | --- |
| 参加校内外的社会实践活动。 *Participated in social practice activities on and off campus.* | ○ | ○ | ○ | ○ | ○ |
| 参加公益或志愿者服务活动。 *Participated in public welfare or volunteer service activities.* | ○ | ○ | ○ | ○ | ○ |
| 参与热点问题的讨论。 *Participated in discussions on social hot-button issues.* | ○ | ○ | ○ | ○ | ○ |
| 向政府机构、学校、媒体等反映意见。 *Expressed opinions to government agencies, schools, or media.* | ○ | ○ | ○ | ○ | ○ |
| 对国家重要会议和法律法规的关注。 *Paid attention to important national conferences and laws and regulations.* | ○ | ○ | ○ | ○ | ○ |

**Total: 69 items**
